# Doxorubicin resistance in breast cancer is mediated via the activation of FABP5/PPARγ and CaMKII signaling pathway

**DOI:** 10.3389/fphar.2023.1150861

**Published:** 2023-07-19

**Authors:** Nan-Nan Chen, Xin-Di Ma, Zhuang Miao, Xiang-Mei Zhang, Bo-Ye Han, Ahmed Ali Almaamari, Jia-Min Huang, Xue-Yan Chen, Yun-Jiang Liu, Su-Wen Su

**Affiliations:** ^1^ The Key Laboratory of Neural and Vascular Biology, The Key Laboratory of New Drug Pharmacology and Toxicology, Department of Pharmacology, Ministry of Education, Hebei Medical University, Shijiazhuang, Hebei, China; ^2^ Breast Center, Hebei Provincial Key Laboratory of Tumor Microenvironment and Drug Resistance, Fourth Hospital of Hebei Medical University, Shijiazhuang, Hebei, China; ^3^ Research Center, Fourth Hospital of Hebei Medical University, Shijiazhuang, Hebei, China

**Keywords:** breast cancer, FABP5, chemoresistance, doxorubicin resistance, calcium, CaMKII, autophagy

## Abstract

Breast cancer is the most prevalent malignancy among women. Doxorubicin (Dox) resistance was one of the major obstacles to improving the clinical outcome of breast cancer patients. The purpose of this study was to investigate the relationship between the FABP signaling pathway and Dox resistance in breast cancer. The resistance property of MCF-7/ADR cells was evaluated employing CCK-8, Western blot (WB), and confocal microscopy techniques. The glycolipid metabolic properties of MCF-7 and MCF-7/ADR cells were identified using transmission electron microscopy, PAS, and Oil Red O staining. FABP5 and CaMKII expression levels were assessed through GEO and WB approaches. The intracellular calcium level was determined by flow cytometry. Clinical breast cancer patient’s tumor tissues were evaluated by immunohistochemistry to determine FABP5 and p-CaMKII protein expression. In the presence or absence of FABP5 siRNA or the FABP5-specific inhibitor SBFI-26, Dox resistance was investigated utilizing CCK-8, WB, and colony formation methods, and intracellular calcium level was examined. The binding ability of Dox was explored by molecular docking analysis. The results indicated that the MCF-7/ADR cells we employed were Dox-resistant MCF-7 cells. FABP5 expression was considerably elevated in MCF-7/ADR cells compared to parent MCF-7 cells. FABP5 and p-CaMKII expression were increased in resistant patients than in sensitive individuals. Inhibition of the protein expression of FABP5 by siRNA or inhibitor increased Dox sensitivity in MCF-7/ADR cells and lowered intracellular calcium, PPARγ, and autophagy. Molecular docking results showed that FABP5 binds more powerfully to Dox than the known drug resistance-associated protein P-GP. In summary, the PPARγ and CaMKII axis mediated by FABP5 plays a crucial role in breast cancer chemoresistance. FABP5 is a potentially targetable protein and therapeutic biomarker for the treatment of Dox resistance in breast cancer.

## 1 Introduction

Breast cancer is already more common than lung cancer as the most prevalent cancer among females, with an expected 2.3 million new cases in 2020. And among female cancer patients, breast cancer (BRCA) accounts for an estimated 0.682 million deaths (15.5%) (Sung et al., 2021). Adriamycin (ADR), also known as doxorubicin (Dox), is an efficient medication for breast cancer, yet the resistance of cancer cells remains a major challenge for achieving satisfactory therapeutic effects (Meredith and Dass, 2016; Zhang et al., 2021a; Pan et al., 2021). Recent research has demonstrated that increased expression of P-glycoprotein (P-GP) (Billington et al., 2018) and differential expression of microRNA and long noncoding RNA (lncRNA) lead to Dox resistance in breast cancer cells (Perez-Anorve et al., 2019; Zheng et al., 2020). Moreover, changes in lipid metabolism are commonly found with tumor growth and acquired drug resistance (Ndiaye et al., 2020).

Fatty acid binding proteins (FABPs), a class of intracellular lipid chaperones, deliver fatty acids (FAs) to specified organelles, including the endoplasmic reticulum, mitochondria, and the nucleus (Trojnar et al., 2019). FABPs mediate the biological characteristics of tumor cells (Lv et al., 2019). As an example, FABP4, a FABP present in mammalian adipocytes, mediates carboplatin resistance in ovarian cancer cells (Mukherjee et al., 2020). Recent research has indicated that FABP5, which stimulates tumor cell proliferation in clear cell renal cell carcinoma, is related to poor survival rates (Lv et al., 2019). FABP5 also serves as a separate indicator of lymph node metastasis in cervical cancer (Zhang et al., 2020).

Intracellular calcium acts as a multifunctional second messenger involved in several physiological and pathological processes. In breast cancer cells, intracellular calcium concentration is intimately related to multidrug resistance-relevant pathways, such as epithelial mesenchymal transition (EMT) and repair of DNA damage. However, few studies investigate the co-interaction between intracellular calcium concentration and medication chemo-resistance (Wen et al., 2016). As a calcium binding protein, soluble resistance-related calcium-binding protein (sorcin, SRI) is correlated to the resistance to chemotherapeutics in cancer cells and has the same amplicon as the P-glycoprotein (P-GP). The overexpression of SRI leads to enhanced resistance to a variety of chemotherapeutic treatments (Colotti et al., 2014). Recently, a new role for glucose-regulated protein 75 (GRP75)-calcium/calmodulin-dependent protein kinase II (CaMKII)-p38 axis suppressing NF-κB-YY1-FAS in rituximab-resistant reverse diffuse large B-cell lymphoma cells has been discovered (Sun et al., 2022). Consequently, we speculated that FABP5 might impact BRCA cell function via the CaMKII/Sequestosome 1 (SQSTM1, P62) signaling pathway, based on the pivotal functions of cellular calcium in tumor cells, particularly BRCA cells.

The relationship between autophagy and tumor metastasis, EMT, apoptosis, and medication resistance is tight (Ye et al., 2017). In the advanced stage of cancers, like breast tumor cells, colon carcinoma cells, and osteosarcoma cells, autophagy is an important cause of medication resistance in cancerous cells and good management of cellular autophagy may increase the susceptibility of therapy (Tian et al., 2019). Based on a recent study, protective autophagy is connected to acquired drug resistance, and inhibiting autophagy has been proposed as an effective cancer therapy (Kim et al., 2018). Autophagy can inhibit the progression of breast cancer and is involved in the development of therapy resistance in breast cancer (Du et al., 2020).

In this study, we determined the function of FABP5 mediated lipid accumulation in the BRCA cell line, and the results suggested that FABP5 regulated the resistance of BRCA cells to Dox. We hoped to present a fresh perspective on the therapy of tumors and a novel approach to breast cancer medication resistance.

## 2 Materials and methods

### 2.1 Cell lines and transfection

MCF-7 cells and MCF-7/ADR cells were purchased from Nanjing KeyGen. Cells were maintained in RPMI-1640 media supplemented with 10% fetal bovine serum (ExCell Bio, Uruguay) and 1% penicillin/streptomycin at 37°C in a humidified 5% CO_2_ atmosphere. MCF-7/ADR cells were cultivated in the presence of a low dose of doxorubicin hydrochloride (1 μg/mL, MCE, China) and passaged once in drug-free media before the tests to retain their resistance characteristic (Wang et al., 2016). MCF-7/ADR cells were transfected with FABP5-specific siRNA (sc-41237, Santa Cruz Biotechnology, USA) and control siRNA with 100 nM lipofectamine 3,000 reagents (L3000008, Invitrogen, USA) according to the instructions provided by the manufacturer. SBFI-26 (GC39236, GlpBio, USA), a FABP5 specific inhibitor, is used to suppress FABP5 expression. In 6 well plates, the cells were grown for observation. FABP5 expression was evaluated by qRT–PCR and WB technique 96 h after the transfection of cells (Lewuillon et al., 2022).

### 2.2 Cell counting Kit-8 assay (CCK8 assay)

MCF-7 cells and MCF-7/ADR cells were placed on 96 well plates (8,000 cells per well). 12 h after seeding, cells were treated with Dox (0.1, 0.3, 1, 3, 10, 30, 100, 300, and 1000 μM) for 48 h or cultured with SBFI-26 (0.1, 0.3, 1, 3, 10, 30, 100, and 300 μM) for 72 h, and cell viability was determined with the CCK8 test (MCE, China) in accordance with manufacturer guidelines. After subtracting the background, relative survival was standardized to the untreated control group. The resistance index of MCF-7/ADR cells to Dox was calculated by the equation: RI = IC_50(MCF-7/ADR cell)_/IC_50(MCF-7 cell)_).

### 2.3 Quantitative real-time PCR (qRT-PCR)

Total RNA was extracted with an appropriate RNA Extraction Kit (Promega, United States) and reverse-transcribed employing random primers and a Reverse Transcription Kit (Promega, United States). Then, the expression levels of target RNAs were evaluated using a Step One Plus Real-Time PCR System and SYBR Green Master Mix (7300R, ABI, United States). GAPDH was employed as an endogenous control, and the fold change was computed using the 2^−ΔΔCT^ technique (Mo et al., 2021). Primer sequences were designed by Sangon Biotech (Table 1).

**TABLE 1:** Primer sequences in qRT-PCR.

Primer name	Forward	Reverse
FABP1	ACA​GTG​GTT​CAG​TTG​GAA​GGT​GAC	GTG​ATT​ATG​TCG​CCG​TTG​AGT​TCG
FABP2	CAG​CAC​TTG​GAA​GGT​AGA​CCG	TCA​TGA​GCT​GCA​AGC​TTC​CTT
FABP3	AGC​ATG​ACC​AAG​CCT​ACC​ACA​ATC	ACC​TTC​CTG​TCA​TCT​GCT​GTT​GTC
FABP4	TCC​TGG​TAC​ATG​TGC​AGA​AAT​GGG	GTG​ACG​CCT​TTC​ATG​ACG​CAT​TC
FABP5	CAG​CTG​GAA​GGA​AGA​TGG​CG	CAT​TGC​GCC​CAT​TTT​TCG​CA
FABP6	GTG​ACG​ATG​ATG​ATG​GTG​GTG​GAG	CCA​TGC​TGC​TGG​GAG​GCT​TTC
FABP7	AGG​CTC​TAG​GCG​TGG​GCT​TTG	TGT​GCT​GAG​AGT​CCT​GAT​GAC​CAC
FABP8	AGC​AAC​AAA​TTC​CTG​GGC​ACC​TG	TTT​GCC​ATC​CCA​TCT​CTG​CAC​TTG
FABP9	GTT​GAG​CCC​TTC​TTG​GGA​ACC​TG	TTT​CAC​TAA​CCC​TGC​CAT​GTT​CCG
FABP12	AGT​TTG​AGG​AAA​TCA​CGC​CAG​GTG	TTC​CTG​CTG​GCT​CTT​CCT​ATA​CCC
CaMK2α	ACC​CAT​CCA​AAC​GCA​TCA​CAG​C	TCT​TCA​GGC​AGT​CCA​CGG​TCT​C
CaMK2β	AAT​CTG​TGA​CCC​AGG​GCT​GAC​C	TGT​GGA​TCG​GCT​TGC​TGT​TCT​TG
CaMK2δ	CCG​GGA​TGG​AAA​GTG​GCA​GAA​TG	TTG​GAA​TAC​AGG​GTG​GCT​TGA​TGG
CaMK2γ	ACC​TCG​TGT​TTG​ACC​TTG​TTA​CCG	GGC​TGG​CAT​CTG​CTT​CAC​TGT​AG

### 2.4 Western blot (WB)

Phosphate-buffered saline (PBS) was used to remove any remaining debris from MCF-7 cells and MCF-7/ADR cells. The cells were lysed in a RIPA lysis solution that contains 1% 0.1 M PMSF and 1% protease inhibitor cocktail. The lysate was centrifuged after being incubated on ice for 30 min. Using the BCA protein assay kit, the quantity of soluble protein in the lysate was quantified, and 30 μg of total protein was added. On 8%–15% SDS-PAGE gels, before transferring samples to a PVDF membrane for 1–2.5 h at 200 mA, samples were separated. The membrane was then blocked at room temperature for 2 h with 5% skim milk in TBS plus 0.1% Tween20 (TBST) and incubated at 4°C for one night with antibodies in TBST. The next day, membranes were treated with a secondary antibody against rabbit or mouse IgG (1:10,000, proteintech, United States) in TBST at room temperature for 2 h. Membranes were rinsed three times with TBST and identified using a Dual color infrared laser imager (LI-COR, United States). Odyssey software was used to examine Western blot pictures, respectively. For membrane labeling, the antibodies include P-GP (1:3,000, HUABIO, China), Multidrug resistance-associated protein (MRP1) (1:3,000, HUABIO, China), Microtubule Associated Protein 1 Light Chain 3 Beta (MAP1LC3B) (1:1,000, ABclonal, China), P62 (1:6,000, MBL, United States), Peroxisome proliferator-activated receptor *γ* (PPARγ, 1:1,000,proteintech, United States), FABP5 (1:500, proteintech, United States), CaMKII (1:500, ABclonal, China), p-CaMKII (1:500, ABclonal, China), and GAPDH (1:10,000, proteintech, United States) (Jeon et al., 2018).

### 2.5 Laser confocal microscopy

Dox is fluorescent by nature and can be stimulated at wavelengths of about λ/nm = 480. Consequently, the Dox concentration in drug-treated cells was assessed by examining the fluorescence intensity of the cells using fluorescence microscopy (Leica, Germany) (Jia et al., 2019). Comparison of fluorescence intensity of MCF-7 and MCF-7/ADR cells using unpaired t-tests.

### 2.6 Transmission electron microscope (TEM)

MCF-7 cells and MCF-7/ADR cells were fixed with 2.5% glutaraldehyde and post-fixed for 2 h with 3% osmium tetroxide (OsO4) in a 6 well plate. The samples were embedded in Epon resin after being dehydrated in ethanol at lowering concentrations and chopped into 100 nm pieces, and then the cells were observed with an 80 kV transmission electron microscope (HT7800, HITACHI, Japan). The morphology of cells was evaluated by TEM.

### 2.7 Periodic acid-schiff (PAS) staining

MCF-7 cells and MCF-7/ADR cells cultured in a 12 well plate were washed with PBS three times, and intracellular glycogen was measured using a PAS kit (G1360, Solarbio, China) in accordance with the manufacturer’s guidelines (Liu et al., 2018). PAS staining assay for detecting intracellular glycogen.

### 2.8 Oil red O staining

After 15 min in paraformaldehyde, MCF-7 cells and MCF-7/ADR cells were incubated with Oil Red O lasting 20 min, rinsed with PBS, stained for 5 min in hematoxylin, then washed in running water. Cells could then be identified by a fluorescent microscope (Olympus Corporation, Japan). ImageJ software was used to examine microscopic pictures of Oil Red O staining (Ning et al., 2022). Oil red O staining assay for intracellular lipid droplets.

### 2.9 Bioinformatics analysis

The BRCA raw data, including FABP5 and CaMKII mRNA expression levels, were obtained from the dataset provided by the GEO (http://www.ncbi.nlm.nih.gov/geo/) to examine the difference in mRNA expression level.

### 2.10 Clinical sample collection and patient characteristics

Breast cancer samples from 10 breast cancer patients, 5 of which were Dox-sensitive and 5 of which were Dox-resistant, were obtained surgically from the Department of Breast Center at the Fourth Hospital of Hebei Medical University. Before surgery, the patients received neoadjuvant chemotherapy. Patient information is in the form (Table 2). All breast cancer patients with hormone receptor positive and female. Written informed consent was gained from each patient. This study was approved by the Hebei Medical University of Medicine’s Fourth Hospital’s Ethics Committee. (Approval number: 2020KY129).

**TABLE 2:** Information for sensitive and resistant patients.

Number	Patient	Gender	Age	ER	PR	HER2	FISH	Ki67	Drug	Group
BRCA01	patient1	female	31	5	0	2+	negative	30	AC-T	sensitive
BRCA02	patient 2	female	38	10	20	2+	negative	30	AC-T	sensitive
BRCA03	patient 3	female	55	90	0	1+		20	AC-T	sensitive
BRCA04	patient 4	female	67	90	70	0		30	AC-T	sensitive
BRCA05	patient 5	female	34	90	3	2+	negative	40	TA	sensitive
BRCA06	patient 1	female	47	60	30	0		50	AC-T	resistant
BRCA07	patient 2	female	47	80	70	0		20	AC-T	resistant
BRCA08	patient 3	female	63	80	70	2+	negative	50	AC-T	resistant
BRCA09	patient 4	female	63	90	70	1+		20	AC-T	resistant
BRCA10	patient 5	female	35	80	70	1+		30	TA	resistant

Doxorubicin hydrochloride liposome injection (A), Cyclophosphamide (C), Docetaxel (T), Estrogen receptor (ER), Progesterone receptors (PR), Human epidermal growth factor receptor-2 (HER2), Fluorescence *In Situ* Hybridization (FISH), Kiel 67 (Ki67).

### 2.11 Immunohistochemistry (IHC)

Immunohistochemistry was performed as described previously (Ryu et al., 2018). Paraffin-embedded breast tumor tissues were sectioned by the pathology laboratory of the Fourth Hospital of Hebei Medical University (Shijiazhuang, Hebei, China) at a thickness of 5 microns. Free-floating slides were cultured in a PBS solution containing 3% H_2_O_2_ (v/v), rinsed in PBS three times, and blocked for 30 min at 37°C with 5% goat serum. The slides were incubated with FABP5(1:200, proteintech, USA) and p-CaMKII (1:200, ABclonal, China) overnight at 4°C. After washing, the slides were cultured for 30 min at 37°C with secondary anti-rabbit IgG (proteintech, USA), and were subsequently scanned via a pathology section scanner (Pannoramic SCAN, 3D HISTECH, Hungary) to observe the expression of FABP5 and p-CaMKII.

### 2.12 Flow cytometry

MCF-7 cells and MCF-7/ADR cells were digested by trypsin and resuspended in PBS, then Fluo-4/AM (F14217, Invitrogen™, USA) fluorescent probe was added at a final concentration of 4 μM. The cells were incubated at 37°C in the dark for 60 min then the Fluo-4/AM working solution was moved out. The cells were washed and resuspended with PBS and then incubated for about 10 min at 37°C in the dark. The intracellular calcium was measured by NovoCyte™ (ACEA Biosciences, USA). Ca^2+^ changes in target cells could be monitored most effectively by employing excitation at λ/nm = 488 blue laser, reception at λ/nm = 525 green light, and the flow cytometry setting for scattered light (Li et al., 2017).

### 2.13 Colony formation

Briefly, in a group, 5,000 MCF-7/ADR cells were seeded into 6 well plates after 96 h of transfection with FABP5 siRNA or si-control (siNC) incubated with or without Dox for 48 h and then transferred to complete media. In another group, 5,000 cells were plated onto 6 well plates and pre-incubated for 24 h with SBFI-26, then incubated with or without Dox for 48 h, and then transferred to complete media. The cells were proliferated for 6 days so that colonies might develop. The colonies were fixed for 15–20 min with 4% paraformaldehyde and dyed with 0.2% crystal violet for 10 min (Wang et al., 2020).

### 2.14 Molecular docking

The 3D structures of P-GP (PDB code: 4Q9H), FABP5 (PDB code: 4LKP), CaMK2β (PDB code: 3BHH), and CaMK2δ (PDB code: 2VN9) were discovered in the PDB database (https://www1.rcsb.org/), and the molecular structures of Dox (PubChem CID: 31703) were acquired from the PubChem website and transferred to PDB format using the freeware Open Babel. The molecular docking studies of P-GP, FABP5, CaMK2β, and CaMK2δ as receptors and Dox as ligand were performed by dehydration and hydrogenation via autodock4.2.6 (OpenEye Scientific Software, USA), and the binding energy was measured and the value less than −1 indicating docking activity. The smaller values indicate stronger docking activity and stronger binding (Bang et al., 2018).

### 2.15 Statistics analysis

An unpaired *t*-test was used when comparing MCF-7 and MCF-7**/**ADR cells. Multiple comparisons between groups were conducted by one-way ANOVA. GraphPad Prism was employed for all analyses (GraphPad Software). Unless otherwise noted, data are presented as means ± SD. All experiments were repeated 3 times or more (*n* ≥ 3). *p* < 0.05 was considered statistical significance. GraphPad Prism 7.0, ImageJ, Adobe Illustrator, and R were used for all statistical analysis and graphing.

## 3 Results

### 3.1 Different characteristics distinguishing MCF-7 cells with MCF-7/ADR cells

**FIGURE 1:**
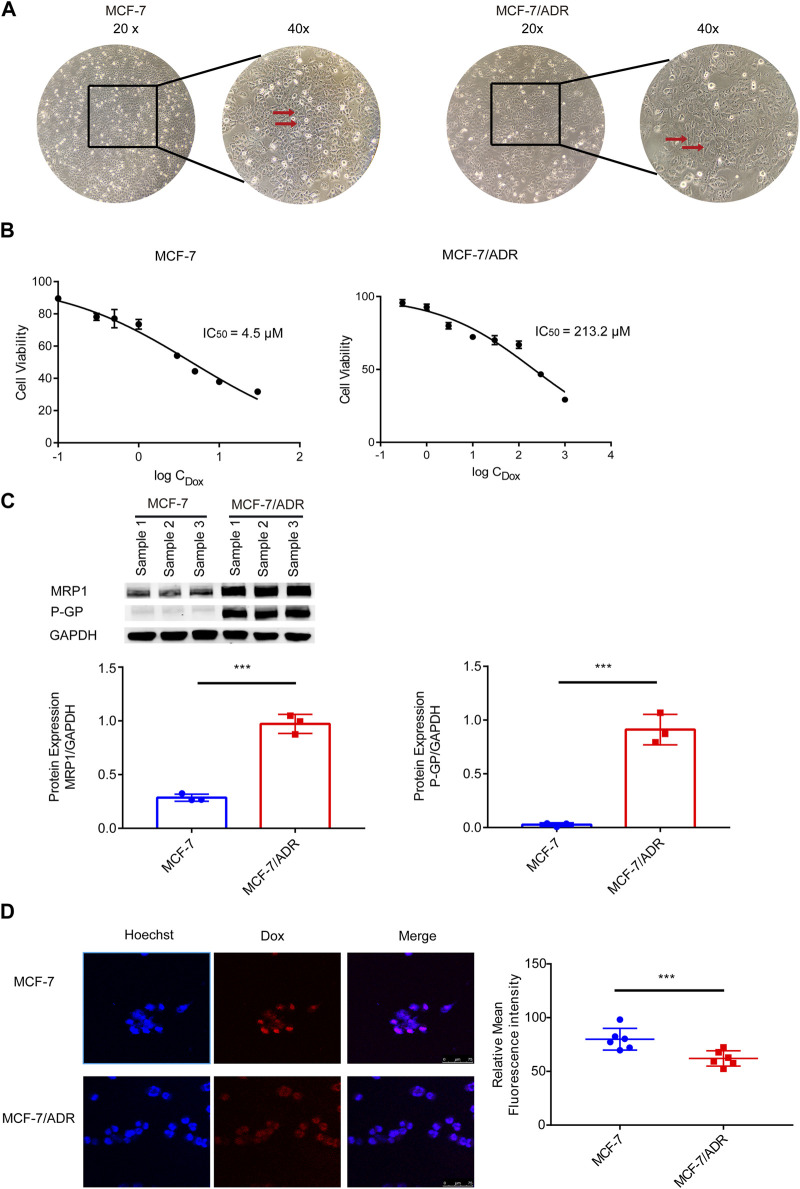
Validation of MCF-7/ADR cell drug resistance qualities. **(A)** MCF-7 cells vary morphologically from MCF-7/ADR cells, MCF-7 cells are spherical shape spheres whereas MCF-7/ADR cells are polyhedral. **(B)** CCK-8 assay was used to determine the IC_50_ values of MCF-7 and MCF-7/ADR cells following treatment of Dox at various doses for 48 h. **(C)** Analysis of the expression of two resistant proteins (MRP1, P-GP) in MCF-7/ADR and MCF-7 cells using WB(*n* = 3). **(D)** After 24 h of treatment with 5 μM Dox, drug accumulation in MCF-7 cells and MCF-7/ADR cells was observed via confocal microscopy. Red fluorescence is the spontaneous color of Dox, blue is the color of the nucleus stained by Hoechst 33,342 and fuchsia is the merge, the intracellular Dox accumulation to be observed, and statistical fluorescence by ImageJ (n = 6), scale 0–75 μm ***p < 0.001 vs. MCF-7 cells.

First, resistant features of MCF-7**/**ADR cells were identified. Differences in morphology between MCF-7 and MCF-7/ADR cells: MCF-7 cells were ellipsoidal with a uniform shape and a distinct three-dimensional structure; MCF-7/ADR cells were polygonal and varied in size (Figure 1A). On MCF-7 cells, the IC_50_ of Dox was 4.5 μM. However, the IC_50_ of Dox was 213.2 μM on MCF-7/ADR cells. The resistance index was 47.4 (Figure 1B). The WB analysis demonstrated that drug resistance-associated proteins, P-GP and MRP1, were considerably differently expressed in MCF-7/ADR cells compared to MCF-7 cells with elevated intracellular expression (Figure 1C). After 24 h of treatment with 5 μM Dox, the intracellular Dox accumulation could be noticed as a fuchsia merging, a substantial decrease was found in drug accumulation in MCF-7/ADR cells compared to MCF-7 cells via confocal microscopy technique (Figure 1D). The above outcomes revealed that MCF-7/ADR cells were indeed MCF-7 resistant cells to Dox.

**FIGURE 2:**
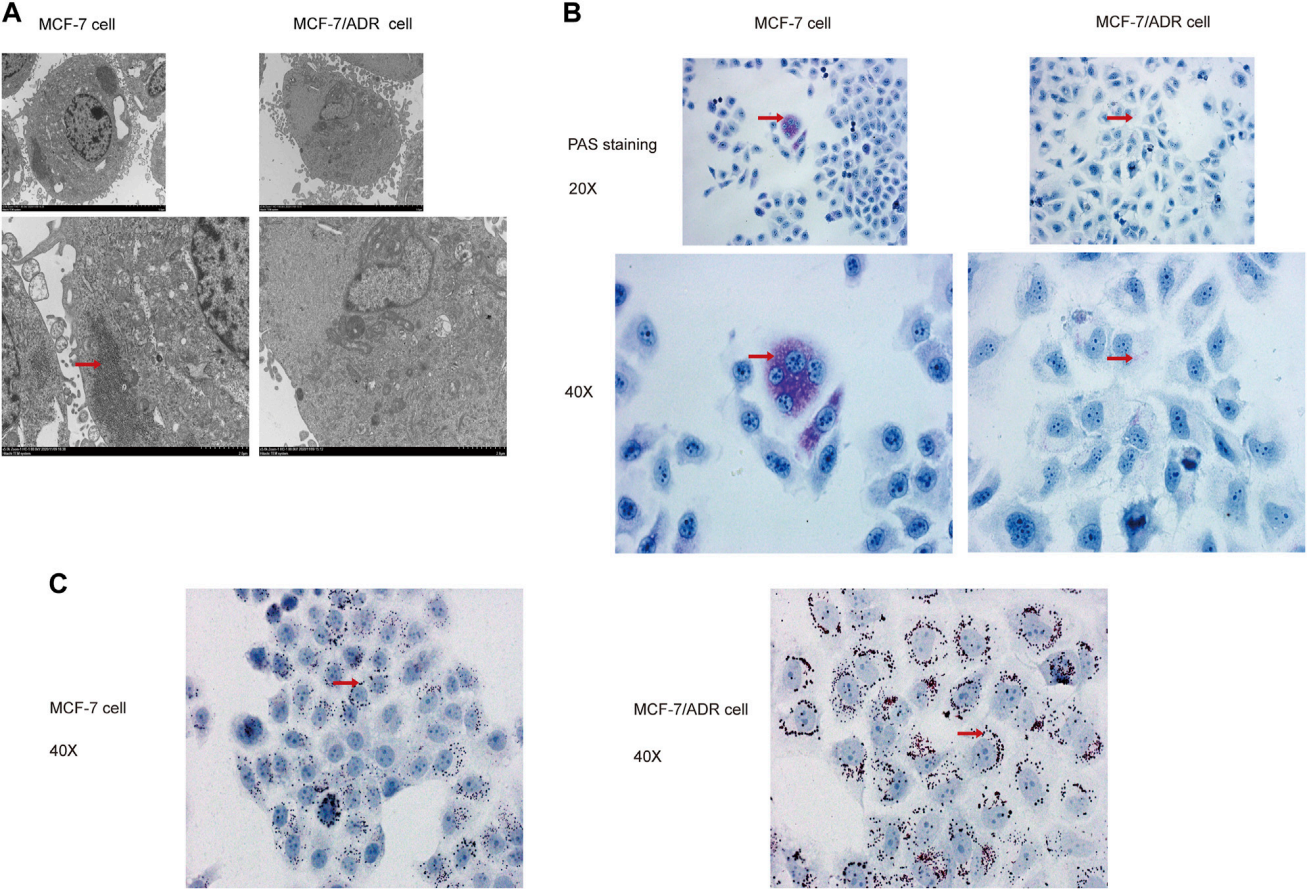
MCF-7 cells and MCF-7/ADR cells had unique glycolipid metabolic profiles. **(A)** Transmission electron microscopy identified the ultrafine structures of MCF-7 and MCF-7/ADR cells, the red arrow points to glycogen, the enlarged image below. **(B)** Micrographs of MCF-7 cells and MCF-7/ADR cells stained with PAS, the red arrow points to glycogen. **(C)** Oil Red O staining of intracellular lipid droplets in MCF-7 and MCF/ADR cells, the red arrow points to lipid droplet.

Then, the superfine structures between MCF-7 cells and MCF-7/ADR cells were observed by transmission electron microscopy. The results demonstrated that MCF-7 cells had considerably more glycogen than MCF-7/ADR cells (Figure 2A). Moreover, glycogen periodic acid-Schiff (PAS) staining revealed that the glycogen content in MCF-7 cells was pretty higher (Figure 2B). However, the oil Red O staining revealed that there were more lipid droplets in MCF-7/ADR cells compared with MCF-7 cells (Figure 2C), indicating that drug resistance may be associated with glycolipid conversion and lipid metabolism.

### 3.2 FABP5 was upregulated in drug-resistant breast cancer cells and tumor tissues

**FIGURE 3:**
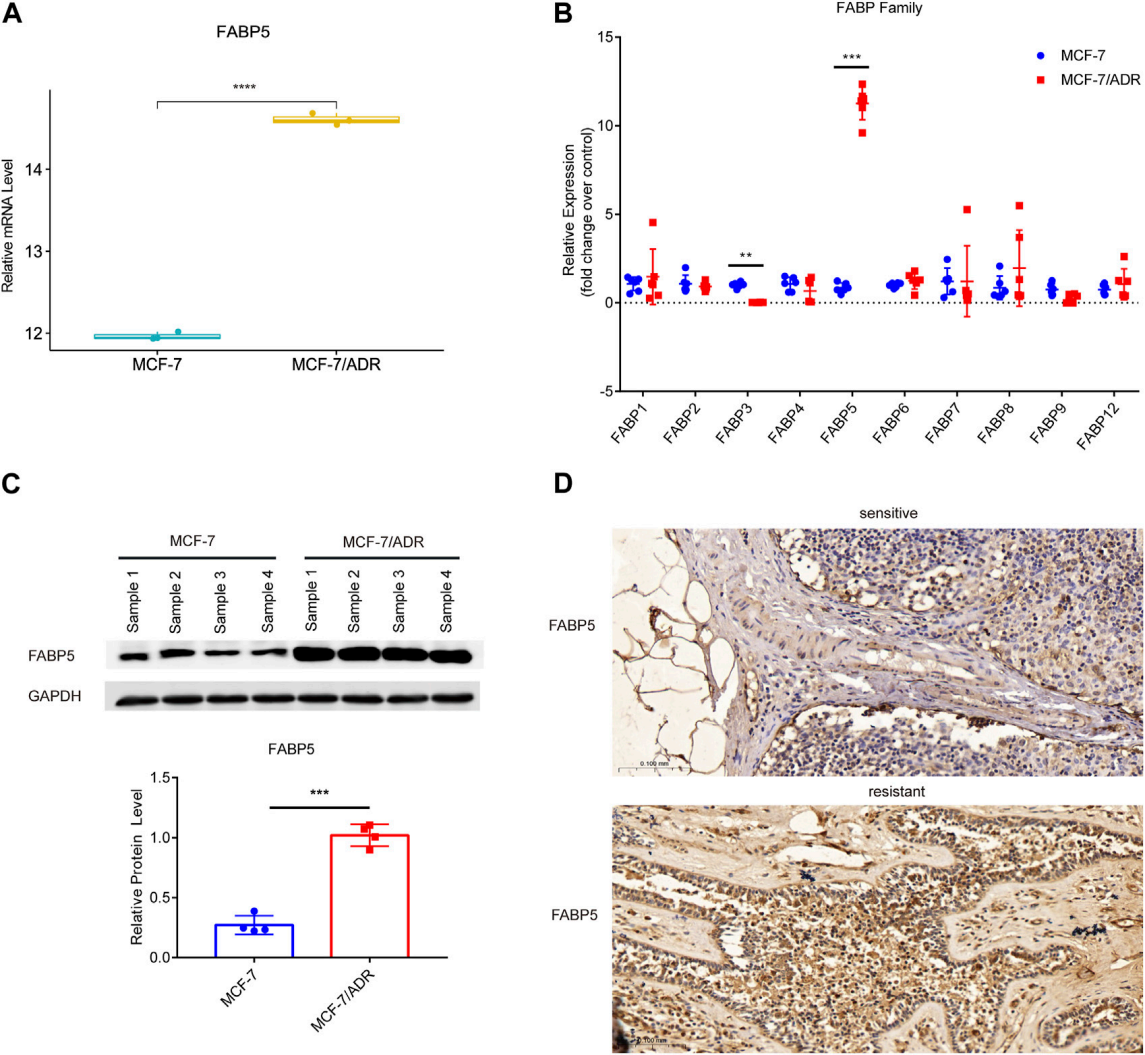
Expression of FABP5 in MCF-7 and MCF-7/ADR cells. **(A)** RNA expression box of FABP5 in the GSE76540 dataset revealed that FABP5 was expressed at a higher level in MCF-7/ADR cells than in MCF-7 cells. **(B)** QRT-PCR analysis of the expression of FABPs mRNAs in MCF-7 and MCF-7/ADR cells (n = 6), and the *Y*-axis represents the fold change over control group (MCF-7). **(C)** WB analysis revealed FABP5 protein expression was increased in MCF-7/ADR cells compared with that in MCF-7 cells (*n* = 4). **(D)** FABP5 expression in breast cancer patients as determined by IHC. Scale bars, 0.1 mm (*n* = 5). **p < 0.01, ***p < 0.001, ****p < 0.0001 vs. MCF-7 cells.

FABPs have a strong affinity for long chain fatty acids (FAs) and control lipid metabolisms in several organs, such as the brain, gut, and liver (Leitao et al., 2018; Reid et al., 2019; Zhang et al., 2021b). FABPs also influence the biological characteristics of tumor cells (Lv et al., 2019). To explore whether the differential expression of FABPs may be induced during the progression of breast cancer chemotherapy resistance, we investigated mRNA microarray data in the GSE76540, a dataset of the Gene Expression Omnibus (GEO) database. RNAs with log_2_FC ≥ 1 and *adj.P.Value* < 0.05 were selected from the microarray data. Compared to MCF-7 cells, MCF-7/ADR cells demonstrated 2,490 upregulated mRNAs and 2,577 downregulated mRNAs. Among them, FABP5 was the only upregulated FABP family member in MCF-7/ADR cells compared to MCF-7 cells (Figure 3A; Supplementary Figure S1A–C).

To confirm the relationship between FABP5 and BRCA resistance, the expression of FABP5 was measured in MCF-7 and MCF-7/ADR cells. qRT-PCR was used to determine the expression levels of 10 FABPs (FABP1, FABP2, FABP3, FABP4, FABP5, FABP6, FABP7, FABP8, FABP9, and FABP12) (Figure 3B). Among them, FABP5 had the greatest level of expression in MCF-7/ADR cells. We did WB assays on two breast cancer cell lines to confirm this result and found that FABP5 was highly expressed in MCF-7/ADR cells (Figure 3C). We collected 10 patients with breast cancer treated routinely with neoadjuvant chemotherapy (NAC): doxorubicin hydrochloride liposome injection, cyclophosphamide, and docetaxel. According to clinical effectiveness, patients were classified as sensitive or resistant, detailed patient information is in Table 2. We evaluated the expression of FABP5 via IHC in 10 breast cancer samples from the Fourth Hospital of Hebei Medical University. The results demonstrated that FABP5 expression was increased in resistant patients than in sensitive individuals, consistent with the performance of the results on cells (Figure 3D; Supplementary Figure S1D). These data indicated that FABP5 may promote Dox resistance *in vitro* and *in vivo*.

### 3.3 Intracellular calcium modulated the resistance of MCF-7/ADR cells to dox

**FIGURE 4:**
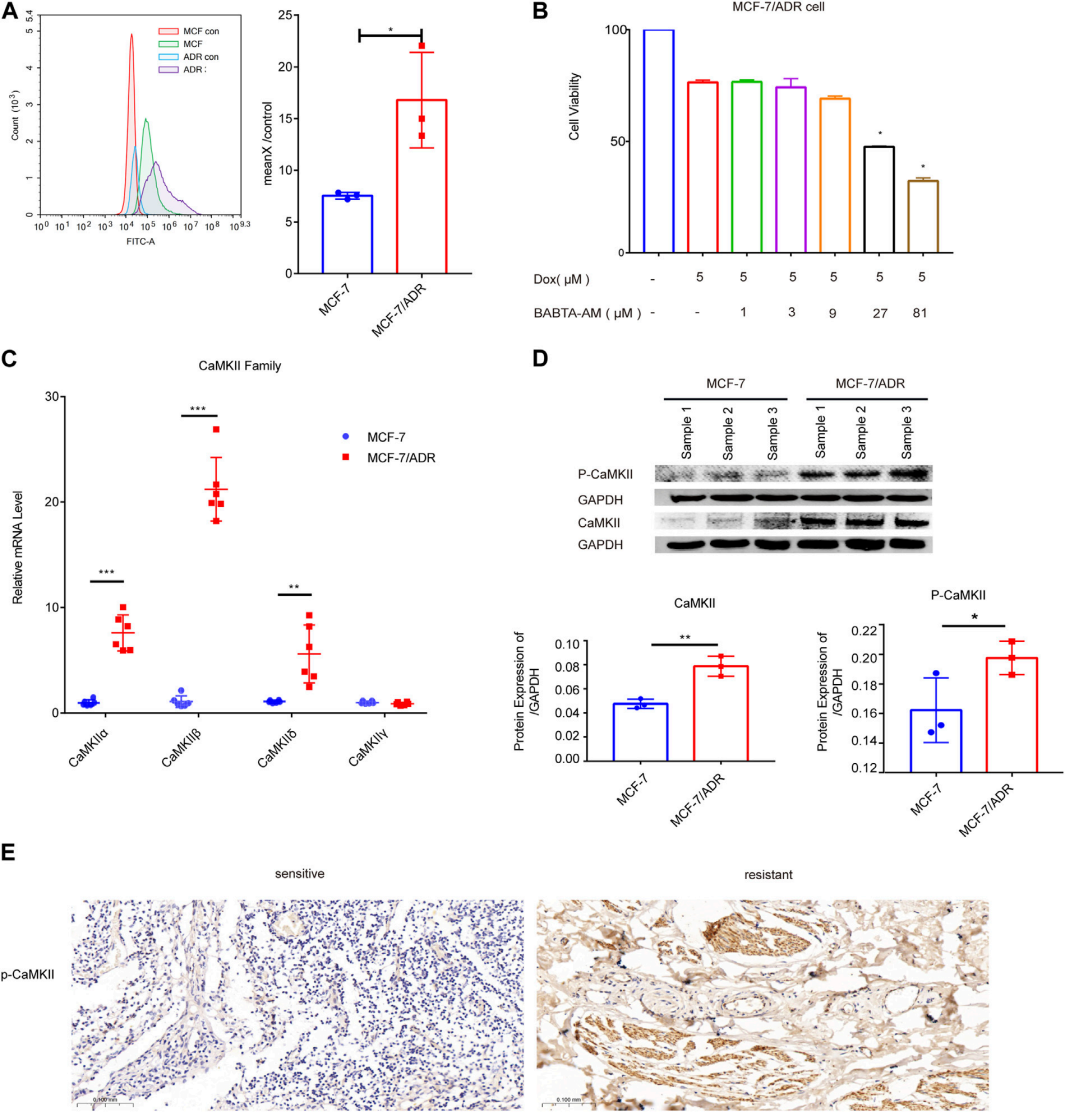
Intracellular calcium in MCF-7 and MCF-7/ADR cells. **(A)** Flow cytometry revealed intracellular calcium accumulation in MCF-7 and MCF-7/ADR cells, with the accumulation being higher in MCF-7/ADR cells. MCF-7 and MCF-7/ADR group were stained with fluo-4 a.m. for 60 min, but MCF-7 con and MCF-7/ADR con group were cellular autofluorescence, MeanX/control means MCF-7/MCF-7 con (*n* = 3). **(B)** MCF-7/ADR cell viability was measured by the CCK-8 method after 5 μM Dox treatment for 24 h with or without preincubation with BABATA-AM for 2 h *p < 0.05 vs*.* Dox (5 μM) group. **(C)** The qRT-PCR analysis of CaMKII family genes expression (*n* = 6). **(D)** The protein expressions of p-CaMKII, CaMKII and GAPDH were analyzed in MCF-7 and MCF-7/ADR cells by the WB technique (*n* = 3). **(E)** p-CaMKII expression in breast cancer patients as determined by IHC. Scale bars, 0.1 mm (*n* = 3). *p < 0.05, **p < 0.01, ***p < 0.001 vs*.* MCF-7 cells.

We used flow cytometry to detect the intracellular calcium levels in MCF-7 and MCF-7/ADR cells, which showed a dramatic increase in calcium expression in MCF-7/ADR cells compared to MCF-7 cells, exceeding MCF-7 cells by 2-fold, MCF-7/ADR cells were able to maintain a sustained intracellular calcium aggregation (Figure 4A). To determine the function of calcium in MCF-7/ADR cell resistance to Dox, cell viability after Dox treatment for 48 h was monitored in the presence or absence of BABTA-AM, a member-permeable intracellular calcium chelator. It was discovered that cell viability reduced proportionally with increasing doses of BABTA-AM. BABTA-AM dose-dependently increased the drug sensitivity of MCF-7/ADR cells to Dox (Figure 4B). The results demonstrated that intracellular calcium is involved in regulating drug resistance in MCF-7/ADR cells. The mRNA expressions of the CaMKII sub-family, except for *CaMKIIγ,* were substantially higher in MCF-7/ADR cells compared to MCF-7 cells, with a 7.40-fold, 19.47-fold, and 5.58-fold increase for *CaMKIIα, CaMKIIβ*, and *CaMKIIδ* respectively (Figure 4C), and protein levels were further confirmed (Figure 4D). In the GSE76540 database, there were no differences for *CaMKIIα* and *CaMKIIβ*, however *CaMKIIδ* and *CaMKIIγ* were substantially different in MCF-7/ADR cells compared to MCF-7 cells (Supplementary Figure S2). We investigated the expression of p-CaMKII via IHC in 3 sensitive patients and 3 resistant patients. The results demonstrated that p-CaMKII expression was increased in resistant patients than in sensitive individuals, agreeing with the performance of the results on cells (Figure 4E; Supplementary Figure S2B). These results indicated that intracellular calcium could promote Dox resistance in MCF-7/ADR cells, and its molecular mechanism might involve the regulation of CaMKII expression.

### 3.4 Inhibition of FABP5 on the drug sensitivity to dox in breast cancer cells

**FIGURE 5:**
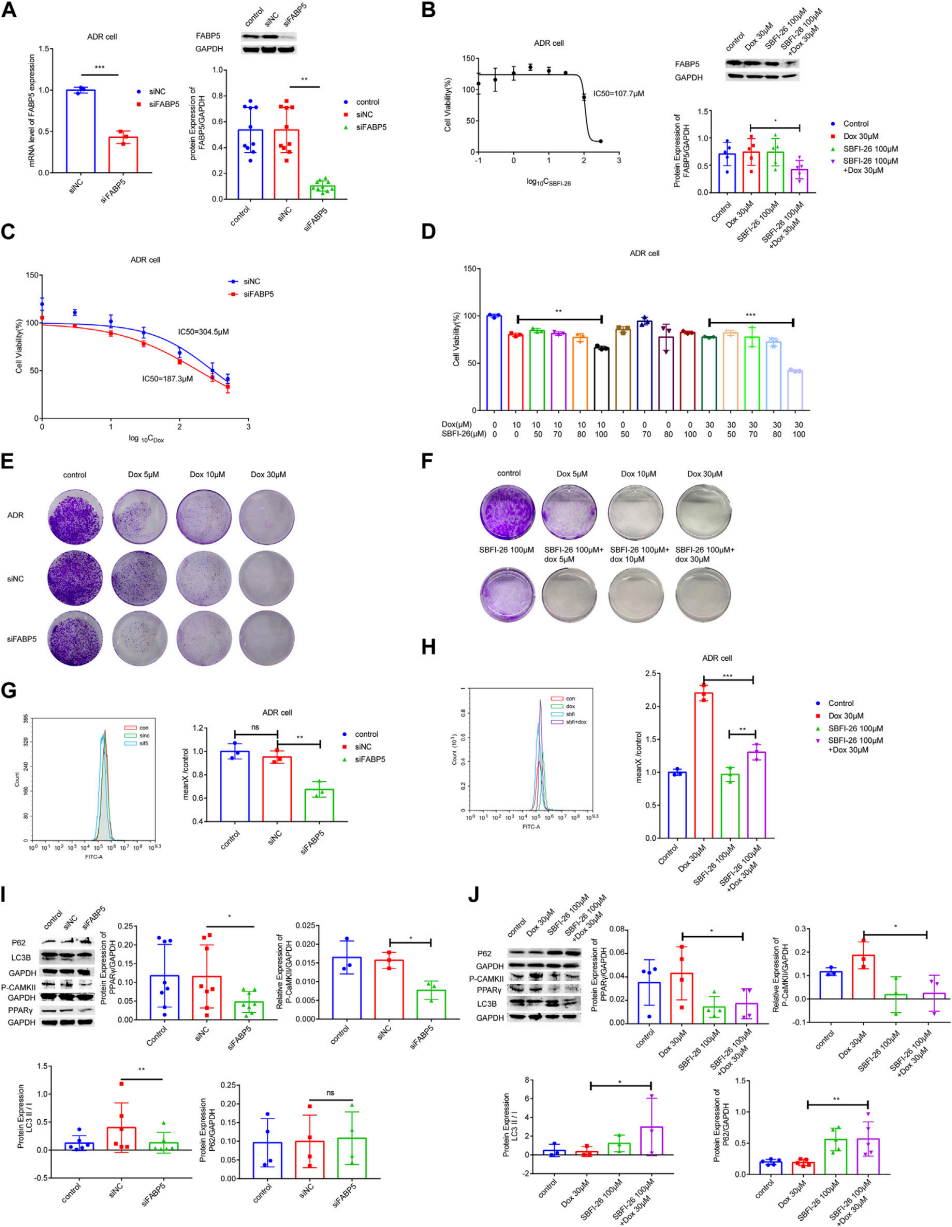
FABP5 affected breast cancer cell drug resistance. **(A)** After the transfection of FABP5 siRNAs for 96 h, the mRNA and protein expression levels of FABP5 in MCF-7/ADR cells were determined. **(B)** Cell viability was examined by CCK-8 assays in cells cultured with FABP5 inhibitor SBFI-26 for 72 h, and the protein expression level of FABP5 in MCF-7/ADR cells was determined. **(C, D)** The drug resistance of cells transfected with FABP5 siRNA and SBFI-26 was evaluated using the CCK-8 test, and resistance was decreased. **(E)** MCF-7/ADR cells transfected with the FABP5 siRNA were seeded onto plates. The colony-forming ability of cells cultured with Dox after transfected with FABP5 siRNAs was determined compared with Dox alone (*n* = 3). **(F)** The colony-forming capacity of cells cultured with SBFI-26 and Dox was reduced compared to Dox (*n* = 3). **(G, H)** Calcium was examined by flow cytometry assay in cells transfected with FABP5 siRNAs and SBFI-26. **(I, J)** WB analysis of p-CaMKII、P62、PPARγ、LC3Ⅰ、LC3Ⅱ and GAPDH in cells transfected with FABP5 siRNAs and SBFI-26. *p < 0.05, **p < 0.01, ***p < 0.001.

To identify the role of FABP5 on the drug sensitivity in breast cancer cells, siFABP5 was transfected in MCF-7/ADR cells for 96 h by using lipofectamine 3,000 or cultured with SBFI-26 for 72 h (an inhibitor of FABP5). The FABP5 silencing effectiveness was assessed by using qRT-PCR and WB techniques. Upon completion of siFABP5 transfection, FABP5 expression was dramatically reduced compared with the si-control (siNC) group (Figure 5A), but different results were obtained for SBFI-26 (Figure 5B). These data suggest that FABP activity, but not protein expression, is decreased by these FABP inhibitors, like SBFI-26 (Wang W. et al., 2021; Lei et al., 2022; Farrell et al., 2023). T hen, we utilized the CCK-8 cytotoxicity assay to determine alterations in the drug sensitivity of cells. After FABP5 was silenced, the cells’ drug sensitivity of MCF-7/ADR cells rose dramatically, and the IC_50_ value was decreased from 304.5μM to 187.3 μM (Figure 5C). The co-incubation of SBFI-26 with Dox resulted in a substantial reduction in cell viability compared to Dox only treatment (Figure 5D). To further confirm the influence of FABP5 on cell proliferation capability, the colony formation experiment showed a substantial decrease in colony formation when Dox was co-cultured with siFABP5 as compared to siNC with Dox (Figure 5E). Compared to Dox alone, the colony forming ability of MCF-7/ADR cells was substantially reduced by simultaneous administration of SBFI-26 and Dox, suggesting that reducing intracellular FABP5 expression could promote the drug sensitivity of cells to Dox (Figure 5F). The results showed that down-regulating the expression of FABP5 could affect cell proliferation and reduce drug resistance.

### 3.5 FABP5 promoted BRCA cell resistance to dox by enhancing intracellular calcium

Next, we used flow cytometry to detect calcium accumulation in cells. The silencing of FABP5 dramatically reduced the accumulation of calcium in cells (Figures 5G,H). After a long period of Dox action, MCF-7/ADR cells could maintain a relatively high level of intracellular calcium, and once the stable state is broken, cell death will occur. Upon completion of siFABP5 transfection, p-CaMKII expression was dramatically reduced compared with the siNC group (Figure 5I), and a similar result was observed with SBFI-26 (Figure 5J), this suggested that FABP5 regulates intracellular calcium by promoting the expression of p-CaMKII, which in turn makes MCF-7/ADR cells resistant to Dox. When FABP5 expression was repressed using siFABP5, the level of PPARγ was lowered relative to the siNC group (Figure 5I). Moreover, when FABP5 expression was inhibited by SBFI-26, the level of PPARγ was decreased relative to the control group, and when SBFI-26 was cocultured with Dox, the level of PPARγ was decreased exclusively relative to Dox (5J). FABP5 influences the Dox resistance of MCF-7/ADR cells by modulating the activation of PPARγ. Compared to siNC, siFABP5 decreased LC3II/Ⅰ, indicating a reduction in autophagic vesicles, but there was no difference in P62, indicating a reduction in autophagic activity (Figure 5I). Our experimental results confirmed that siFABP5 leads to a reduction in LC3II/I and thus in the production of autophagic vesicles. Thus, we predict that siFABP5 increases breast cancer cell susceptibility to chemotherapy. Compared to Dox administration alone, the simultaneous administration of SBFI-26 and Dox increased LC3II/Ⅰ, suggesting an increase in autophagic vesicles, and P62, showing suppression of the autophagic vesicle degradation process (Figure 5J). In addition, we evaluated the expression of autophagic proteins in MCF-7 and MCF-7/ADR cells and observed a substantial increase in LC3II/Ⅰ and a decrease in P62 in drug-resistant cells relative to sensitive cells, indicating that MCF-7/ADR cells were more autophagic (Supplementary Figure S3). The preceding data suggested that decreased intracellular FABP5 expression inhibits cellular resistance to Dox by regulating intracellular autophagy.

### 3.6 Molecular docking model with potential targets

**FIGURE 6:**
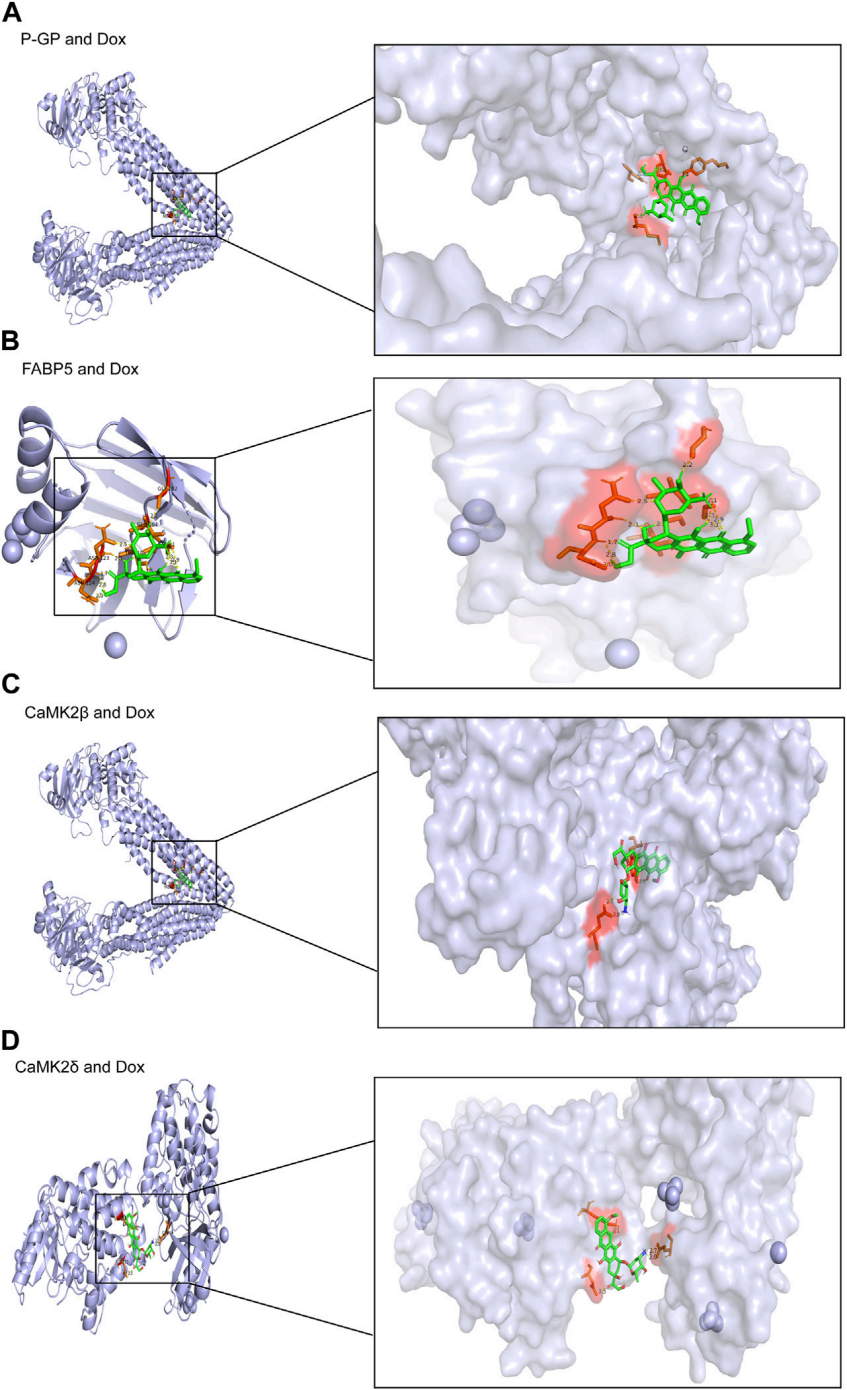
Molecular docking results of Dox and proteins. **(A)** Dox (PubChem CID: 31703) bound to P-GP (PDB code: 4Q9H). **(B)** Dox (PubChem CID: 31703) bound to FABP5 (PDB code: 4LKP). **(C)** Dox (PubChem CID: 31703) bound to CaMK2β (PDB code: 3BHH). **(D)** Dox (PubChem CID: 31703) bound to CaMK2δ (PDB code: 2VN9).

To verify the expected outcome, we presented a molecular docking model containing probable targets. AutoDock4.2.6 provides a semi-empirical free energy calculation method for determining the energy match between a receptor and ligand. The ligand-receptor interaction calculation is primarily based on the molecular mechanics method and consists of five components: van der Waals forces, electrostatic interactions, hydrogen bonding, desolvation, and torsional entropy change. Dox has many binding sites to the protein, and when the binding energies of the various sites were evaluated, it was discovered that Dox had greater binding energy to FABP5 (dock score varied from −6.00 to −8.78 kcal/mol) than to P-GP (dock score varied from −1.47 to −3.01 kcal/mol). Moreover, the binding energies of Dox to CaMK2β (dock score varied from −2.85 to −4.89 kcal/mol) and Dox to CaMK2δ (dock score varied from −3.68 to −4.60 kcal/mol) were determined to be moderately high (Figures 6A–D; Table 3). According to the lowest binding energy (ΔG_binding_), we evaluated the binding form between Dox with the proteins (Table 4). These results demonstrated that FABP5 mainly bonds to Dox with the van der Waals forces and hydrogen bonding and desolvation, which make Dox can dissociate with FABP5 easily, indicating that FABP5 may play a crucial role in the mechanism of Dox resistance.

**TABLE 3:** Summary of docking analysis of Dox with various proteins from Auto Dock Vina scores.

PDB/Dox	P-GP (kcal/mol)	FABP5 (kcal/mol)	CaMK2β (kcal/mol)	CaMK2δ (kcal/mol)
Dox 1	−3.01	−8.78	−4.89	−4.60
Dox 2	−2.91	−7.34	−4.05	−4.32
Dox 3	−2.74	−6.99	−3.67	−4.26
Dox 4	−2.18	−6.96	−3.45	−4.22
Dox 5	−2.16	−6.69	−3.43	−4.19
Dox 6	−1.67	−5.90	−3.42	−4.16
Dox 7	−1.54	−6.65	−2.90	−3.91
Dox 8	−1.47	−6.00	−2.85	−3.68

**TABLE 4:** Various energies of the complexes obtained by Auto Dock docking.

PDB/Dox	ΔG_binding_ (kcal/mol)	ΔE_1_ (kcal/mol)	ΔE_2_ (kcal/mol)	ΔE_3_ (kcal/mol)	ΔE_4_ (kcal/mol)
P-GP	−3.01	−6.51	0.21	3.28	−3.99
FABP5	−8.78	−9.66	−2.13	3.02	−3.02
CaMK2β	−4.89	−6.35	−1.82	3.28	−6.28
CaMK2δ	−4.60	−5.21	−2.41	3.02	−5.22

ΔE_1_: vdW + Hbond + desolv energy; ΔE_2_: electrostatic internal; ΔE_3_: torsional energy; ΔE_4_: unbound energy.

## 4 Discussion and conclusion

**FIGURE 7:**
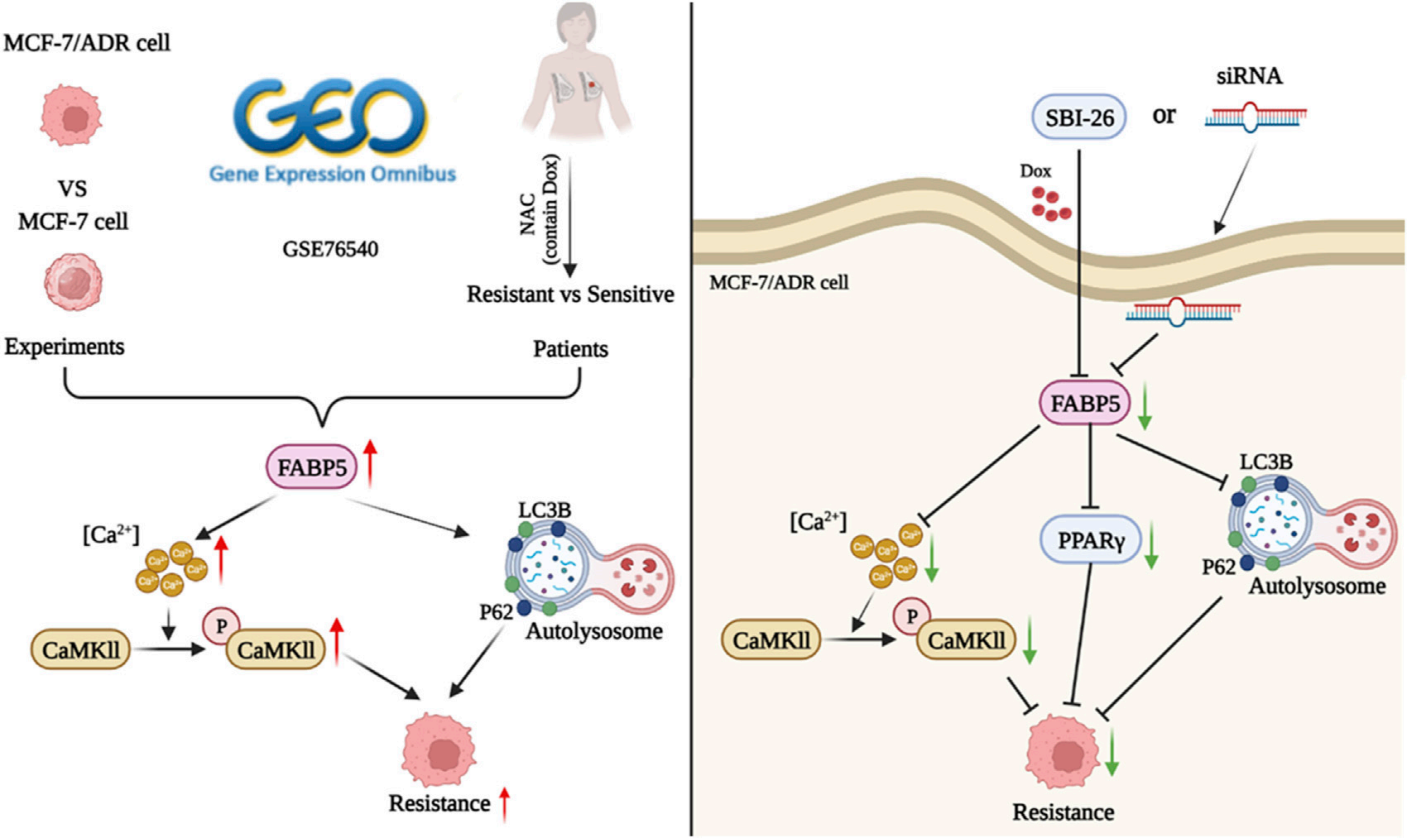
The role of FABP5 in breast cancer resistance to doxorubicin.

We compared the properties of MCF-7 and MCF-7/ADR cells in considerable detail, including morphology, protein expression, cellular ultrastructure, and variations in lipid metabolism. The expression of FABP5 and intracellular calcium, which were predicted to be potentially implicated in cellular drug resistance, was found to be considerably different. To further substantiate the role of FABP5 and enhance the validity of our findings, we sought to ascertain its clinical relevance to potentially identify new approaches for therapeutic intervention. We analyzed tissue samples from breast cancer patients given the adjuvant chemotherapy regimen containing doxorubicin. The IHC results demonstrated that FABP5 and p-CaMKII expression were increased in resistant patients than in sensitive individuals, agreeing with the performance of the results on cells. Therefore, we inhibited intracellular FABP5 expression and observed modifications in cellular resistance to Dox. Following inhibition of FABP5 expression in MCF-7/ADR cells, no significant changes in CaMKII protein were observed, and this protein was not subsequently studied. In addition, in MCF-7/ADR cells, inhibition of protein expression of FABP5 by siRNA or using FABP5 inhibitor resulted in decreased PPARγ and p-CaMKII, a reduction in intracellular calcium, and different changes in the intracellular autophagy proteins LC3II/I and P62, suggesting that FABP5 can further regulate autophagy by regulating PPARγ and p-CaMKII, which in turn affects cellular resistance to Dox (Figure 7).

BRCA is prevalent cancer that mostly affects women all around the world and has different presentations (Barzaman et al., 2020). Chemotherapy is a standard treatment, and epirubicin and doxorubicin are the most often administered medicines for breast cancers. Nevertheless, its clinical application is limited due to its susceptibility to resistance (Akram et al., 2017). Through the colony formation experiment, we found that when the cells were few and scattered, a low dose of Dox could kill them, but when the tumor cells were many and aggregated, even 30 μM of Dox had little effect on them. With continued stimulation of the drug, the cells underwent continuous changes but did not apoptosis, creating drug resistance. Yet, cancer cell resistance has appeared as a serious obstacle to Dox-based treatment. Autophagy modulation (either activation or inhibition) has been found to overcome or partially reverse Dox resistance in a range of cancer types, indicating that this might be a potential strategy for overcoming Dox resistance in the treatment of cancer (Chen et al., 2018).

Autophagy exhibited dualistic behavior, functioning as both a cell survival process and a cell death mechanism. Several types of malignancies acquire chemoresistance via the enhancement of autophagic flux (Faraone et al., 2020). Some researchers had demonstrated that blocking the merger of lysosomes and autophagosomes increased the susceptibility of BRCA cells to chemotherapy by releasing Dox from lysosomes into the nucleus (Wen et al., 2020). Autophagy is related to the formation of lipid droplets, which are also regulated by FABP (Tan et al., 2017). Our previous experiments demonstrated that both lipid droplets and FABP5 were more abundant in MCF-7/ADR cells than in MCF-7 cells, and hence we investigated the intracellular autophagy. Meantime, our experimental results confirmed that siFABP5 decreased LC3II/I, leading to the production of autophagic vesicles, but does not affect P62, indicating a decrease in autophagic activity. Our experimental results demonstrated that simultaneous administration of SBFI-26 and Dox increased LC3II/I, implying an increase in autophagic vesicles, and elevated P62, a rise in autophagic degradation, meaning a decrease in overall autophagy levels, in comparison to Dox administration alone. All of the preceding results suggest that reducing FABP5 expression inhibits intracellular autophagy and improved Dox sensitivity. Chloroquine, an autophagolysosomal formation inhibitor, produced analogous results in MCF-7/ADR cells, reestablishing their sensitivity to Dox (Guo et al., 2016). It is been revealed that autophagy can be regulated by calcium to promote cell survival (Sukumaran et al., 2021).

In addition, CaMKII is likely the main essential regulator of autophagy in response to intracellular Ca^2+^ elevation. Ovarian cancer’s resistance to paclitaxel was boosted by SRI, and its malignant growth was accelerated (Zhang J. et al., 2021). CaMKII and protein kinase A had been shown to interact with SRI in a Ca^2+^-dependent manner (Wang Y. et al., 2021). Ca^2+^ dependent CaMKII activation played a pivotal role in Dox induced cardiotoxicity by increasing apoptosis in neonatal cardiomyocytes (Tscheschner et al., 2019). According to our findings, MCF-7 cells became resistant to Dox due to elevated levels of p-CaMKII caused by calcium buildup inside the cells.

FABPs are fatty acid binding proteins belonging to a class of cytosolic proteins with a low molecular mass (around 14–15 KD). FABPs regulate the enzyme activities and cytoplasmic accumulation of lipid droplets (Furuhashi and Hotamisligil, 2008). By comparing the variations in mRNA levels between MCF-7 cells and MCF-7/ADR cells, we established that FABP5 was substantially elevated in MCF-7/ADR cells than in MCF-7 cells. In addition to searching the GEO database GSE76540 dataset, FABP5 expression increased in drug-resistant cells than in sensitive cells. Furthermore, we examined the GSE155478 (Zhang et al., 2021a), GSE24460 (Calcagno et al., 2010), and GSE141698 datasets and found the precise same results as formerly (Supplementary Figure S4). In addition, resveratrol reverts FABP5/PPAR-β/δ/-mediated retinoic acid (RA) signaling, which allows RA-resistant anaplastic thyroid cancer cells to become sensitive (Li et al., 2018). Researchers in the study found that the anticancer medication doxorubicin properly suppressed TNBC recurrence and lung metastasis via decreasing the (FABP4/FABP5)/EET dynamics and levels (Apaya et al., 2020). Our results showed that inhibition of FABP5 greatly enhanced cellular drug sensitivity and reduced autophagic activity in MCF/ADR cells. Furthermore, silencing of FABP5 dramatically decreased calcium buildup in cells. The results showed that FABP5 regulated the Dox resistance of breast cancer cells by modulating intracellular calcium expression. And we provide a potential route for clinical chemotherapy that enables the extended use of doxorubicin. To further demonstrate the function of FABP5, we performed molecular docking experiments and the result revealed that FABP5 bound to Dox more strongly than P-GP. P-GP is the most characterized efflux protein pump of the adenosine triphosphate binding cassette superfamily of transporters (Martins et al., 2019). Similar to the P-GP, SRI is a calcium binding protein that has been linked to cancer cell resilience to chemotherapeutics. When overexpressed, SRI causes cells to be more resistant to a wide range of chemotherapy agents (Colotti et al., 2014). In addition, FABP5 is fatty acid binding protein, with a stronger binding ability with Dox than that with P-GP. We also found that the expressions of FABP5 were increased in MCF-7/ADR cells than those in this parent MCF-7 cell. It may act as a P-GP like protein and can pump Dox out of the cell. Further experiments are needed to explore this problem in the future.

In conclusion, FABP5 enhanced breast cancer’s resistance to Dox through PPARγ and the calcium signaling pathway. The FABP5/PPARγ and FABP5/CaMKII axes provided insight into the processes of breast cancer treatment resistance and theoretical support for the hunt for therapeutic targets.

## Data Availability

The original contributions presented in the study are included in the article/Supplementary Material, further inquiries can be directed to the corresponding authors.

## References

[B1] Akram M. Iqbal M. Daniyal M. Khan A. U. (2017). Awareness and current knowledge of breast cancer. Biol. Res. 50 (1), 33. 10.1186/s40659-017-0140-9 28969709 PMC5625777

[B2] Apaya M. K. Hsiao P. W. Yang Y. C. Shyur L. F. (2020). Deregulating the CYP2C19/epoxy-eicosatrienoic acid-associated FABP4/FABP5 signaling network as a therapeutic approach for metastatic triple-negative breast cancer. Cancers (Basel) 12 (1), 199. 10.3390/cancers12010199 31941087 PMC7016875

[B3] Bang E. Noh S. G. Ha S. Jung H. J. Kim D. H. Lee A. K. (2018). Evaluation of the novel synthetic tyrosinase inhibitor (Z)-3-(3-bromo-4-hydroxybenzylidene)thiochroman-4-one (MHY1498) *in vitro* and in silico. Molecules 23 (12), 3307. 10.3390/molecules23123307 30551624 PMC6321646

[B4] Barzaman K. Karami J. Zarei Z. Hosseinzadeh A. Kazemi M. H. Moradi-Kalbolandi S. (2020). Breast cancer: Biology, biomarkers, and treatments. Int. Immunopharmacol. 84, 106535. 10.1016/j.intimp.2020.106535 32361569

[B5] Billington S. Ray A. S. Salphati L. Xiao G. Chu X. Humphreys W. G. (2018). Transporter expression in noncancerous and cancerous liver tissue from donors with hepatocellular carcinoma and chronic hepatitis C infection quantified by LC-MS/MS proteomics. Drug Metab. Dispos. 46 (2), 189–196. 10.1124/dmd.117.077289 29138286 PMC5776333

[B6] Calcagno A. M. Salcido C. D. Gillet J. P. Wu C. P. Fostel J. M. Mumau M. D. (2010). Prolonged drug selection of breast cancer cells and enrichment of cancer stem cell characteristics. J. Natl. Cancer Inst. 102 (21), 1637–1652. 10.1093/jnci/djq361 20935265 PMC2970576

[B7] Chen C. Lu L. Yan S. Yi H. Yao H. Wu D. (2018). Autophagy and doxorubicin resistance in cancer. Anticancer Drugs 29 (1), 1–9. 10.1097/cad.0000000000000572 29099416

[B8] Colotti G. Poser E. Fiorillo A. Genovese I. Chiarini V. Ilari A. (2014). Sorcin, a calcium binding protein involved in the multidrug resistance mechanisms in cancer cells. Molecules 19 (9), 13976–13989. 10.3390/molecules190913976 25197934 PMC6271628

[B9] Du J. Li J. Song D. Li Q. Li L. Li B. (2020). Matrine exerts anti-breast cancer activity by mediating apoptosis and protective autophagy via the AKT/mTOR pathway in MCF-7 cells. Mol. Med. Rep. 22 (5), 3659–3666. 10.3892/mmr.2020.11449 33000249 PMC7533454

[B10] Faraone I. Labanca F. Ponticelli M. De Tommasi N. Milella L. (2020). Recent clinical and preclinical studies of hydroxychloroquine on RNA viruses and chronic diseases: A systematic review. Molecules 25 (22), 5318. 10.3390/molecules25225318 33202656 PMC7696151

[B11] Farrell M. Fairfield H. Karam M. D'Amico A. Murphy C. S. Falank C. (2023). Targeting the fatty acid binding proteins disrupts multiple myeloma cell cycle progression and MYC signaling. Elife 12, e81184. 10.7554/eLife.81184 36880649 PMC9995119

[B12] Furuhashi M. Hotamisligil G. S. (2008). Fatty acid-binding proteins: Role in metabolic diseases and potential as drug targets. Nat. Rev. Drug Discov. 7 (6), 489–503. 10.1038/nrd2589 18511927 PMC2821027

[B13] Guo B. Tam A. Santi S. A. Parissenti A. M. (2016). Role of autophagy and lysosomal drug sequestration in acquired resistance to doxorubicin in MCF-7 cells. BMC Cancer 16 (1), 762. 10.1186/s12885-016-2790-3 27687594 PMC5043608

[B14] Jeon K. H. Yu H. V. Kwon Y. (2018). Hyperactivated m-calpain affects acquisition of doxorubicin resistance in breast cancer cells. Biochim. Biophys. Acta Gen. Subj. 1862 (5), 1126–1133. 10.1016/j.bbagen.2018.02.002 29425806

[B15] Jia Y. Wang C. Zheng J. Lin G. Ni D. Shen Z. (2019). Novel nanomedicine with a chemical-exchange saturation transfer effect for breast cancer treatment *in vivo* . J. Nanobiotechnology 17 (1), 123. 10.1186/s12951-019-0557-0 31847857 PMC6918642

[B16] Kim T. W. Lee S. Y. Kim M. Cheon C. Jang B. H. Shin Y. C. (2018). DSGOST regulates resistance via activation of autophagy in gastric cancer. Cell Death Dis. 9 (6), 649. 10.1038/s41419-018-0658-y 29844404 PMC5974125

[B17] Lei Q. Yu Z. Li H. Cheng J. Wang Y. (2022). Fatty acid-binding protein 5 aggravates pulmonary artery fibrosis in pulmonary hypertension secondary to left heart disease via activating wnt/β-catenin pathway. J. Adv. Res. 40, 197–206. 10.1016/j.jare.2021.11.011 36100327 PMC9481948

[B18] Leitao J. Carvalhana S. Silva A. P. Velasco F. Medeiros I. Alves A. C. (2018). No evidence for lower levels of serum vitamin D in the presence of hepatic steatosis. A study on the Portuguese general population. Int. J. Med. Sci. 15 (14), 1778–1786. 10.7150/ijms.26586 30588203 PMC6299420

[B19] Lewuillon C. Guillemette A. Titah S. Shaik F. A. Jouy N. Labiad O. (2022). Involvement of ORAI1/SOCE in human AML cell lines and primary cells according to ABCB1 activity, LSC compartment and potential resistance to ara-C exposure. Int. J. Mol. Sci. 23 (10), 5555. 10.3390/ijms23105555 35628366 PMC9141756

[B20] Li X. You M. Liu Y. J. Jin P. P. Zhou R. (2017). Reversal of the apoptotic resistance of non-small-cell lung carcinoma towards TRAIL by natural product toosendanin. Sci. Rep. 7, 42748. 10.1038/srep42748 28209994 PMC5314365

[B21] Li Y. T. Tian X. T. Wu M. L. Zheng X. Kong Q. Y. Cheng X. X. (2018). Resveratrol suppresses the growth and enhances retinoic acid sensitivity of anaplastic thyroid cancer cells. Int. J. Mol. Sci. 19 (4), 1030. 10.3390/ijms19041030 29596381 PMC5979404

[B22] Liu X. Wang J. Gao L. Jiao Y. Liu C. (2018). Maternal protein restriction induces alterations in hepatic unfolded protein response-related molecules in adult rat offspring. Front. Endocrinol. (Lausanne) 9, 676. 10.3389/fendo.2018.00676 30524373 PMC6262354

[B23] Lv Q. Wang G. Zhang Y. Han X. Li H. Le W. (2019). FABP5 regulates the proliferation of clear cell renal cell carcinoma cells via the PI3K/AKT signaling pathway. Int. J. Oncol. 54 (4), 1221–1232. 10.3892/ijo.2019.4721 30968158 PMC6411348

[B24] Martins E. Silva V. Lemos A. Palmeira A. Puthongking P. Sousa E. (2019). Newly synthesized oxygenated xanthones as potential P-glycoprotein activators: *In vitro*, *ex vivo*, and in silico studies. Molecules 24 (4), 707. 10.3390/molecules24040707 30781374 PMC6412186

[B25] Meredith A. M. Dass C. R. (2016). Increasing role of the cancer chemotherapeutic doxorubicin in cellular metabolism. J. Pharm. Pharmacol. 68 (6), 729–741. 10.1111/jphp.12539 26989862

[B26] Mo Y. Wang Y. Zhang S. Xiong F. Yan Q. Jiang X. (2021). Circular RNA circRNF13 inhibits proliferation and metastasis of nasopharyngeal carcinoma via SUMO2. Mol. Cancer 20 (1), 112. 10.1186/s12943-021-01409-4 34465340 PMC8406723

[B27] Mukherjee A. Chiang C. Y. Daifotis H. A. Nieman K. M. Fahrmann J. F. Lastra R. R. (2020). Adipocyte-induced FABP4 expression in ovarian cancer cells promotes metastasis and mediates carboplatin resistance. Cancer Res. 80 (8), 1748–1761. 10.1158/0008-5472.CAN-19-1999 32054768 PMC10656748

[B28] Ndiaye H. Liu J. Y. Hall A. Minogue S. Morgan M. Y. Waugh M. G. (2020). Immunohistochemical staining reveals differential expression of ACSL3 and ACSL4 in hepatocellular carcinoma and hepatic gastrointestinal metastases. Biosci. Rep. 40 (4). 10.1042/BSR20200219 PMC7198044 32286604

[B29] Ning Z. Guo X. Liu X. Wang A. Wang X. (2022). USP22 regulates lipidome accumulation by stabilizing PPARγ in hepatocellular carcinoma. Nat. Commun. 13 (1), 2187. 10.1038/s41467-022-29846-9 35449157 PMC9023467

[B30] Pan X. Hong X. Li S. Meng P. Xiao F. (2021). METTL3 promotes adriamycin resistance in MCF-7 breast cancer cells by accelerating pri-microRNA-221-3p maturation in a m6A-dependent manner. Exp. Mol. Med. 53 (1), 91–102. 10.1038/s12276-020-00510-w 33420414 PMC8080609

[B31] Perez-Anorve I. X. Gonzalez-De la Rosa C. H. Soto-Reyes E. Beltran-Anaya F. O. Del Moral-Hernandez O. Salgado-Albarran M. (2019). New insights into radioresistance in breast cancer identify a dual function of miR-122 as a tumor suppressor and oncomiR. Mol. Oncol. 13 (5), 1249–1267. 10.1002/1878-0261.12483 30938061 PMC6487688

[B32] Reid M. J. A. Ma Y. Golovaty I. Okello S. Sentongo R. Feng M. (2019). Association of gut intestinal integrity and inflammation with insulin resistance in adults living with HIV in Uganda. AIDS Patient Care STDS 33 (7), 299–307. 10.1089/apc.2019.0032 31188016 PMC6602108

[B33] Ryu Y. K. Park H. Y. Go J. Choi D. H. Kim Y. H. Hwang J. H. (2018). Metformin inhibits the development of L-DOPA-induced dyskinesia in a murine model of Parkinson's disease. Mol. Neurobiol. 55 (7), 5715–5726. 10.1007/s12035-017-0752-7 29039022

[B34] Sukumaran P. Nascimento Da Conceicao V. Sun Y. Ahamad N. Saraiva L. R. Selvaraj S. (2021). Calcium signaling regulates autophagy and apoptosis. Cells 10 (8), 2125. 10.3390/cells10082125 34440894 PMC8394685

[B35] Sun Q. Ye Y. Gui A. Sun X. Xie S. Zhan Y. (2022). MORTALIN-Ca(2+) axis drives innate rituximab resistance in diffuse large B-cell lymphoma. Cancer Lett. 537, 215678. 10.1016/j.canlet.2022.215678 35447282

[B36] Sung H. Ferlay J. Siegel R. L. Laversanne M. Soerjomataram I. Jemal A. (2021). Global cancer statistics 2020: GLOBOCAN estimates of incidence and mortality worldwide for 36 cancers in 185 countries. CA Cancer J. Clin. 71 (3), 209–249. 10.3322/caac.21660 33538338

[B37] Tan Q. Q. Liu W. Zhu F. Lei C. L. Hahn D. A. Wang X. P. (2017). Describing the diapause-preparatory proteome of the beetle colaphellus bowringi and identifying candidates affecting lipid accumulation using isobaric tags for mass spectrometry-based proteome quantification (iTRAQ). Front. Physiol. 8, 251. 10.3389/fphys.2017.00251 28491041 PMC5405119

[B38] Tian Y. D. Lin S. Yang P. T. Bai M. H. Jin Y. Y. Min W. L. (2019). Saikosaponin-d increases the radiosensitivity of hepatoma cells by adjusting cell autophagy. J. Cancer 10 (20), 4947–4953. 10.7150/jca.30286 31598167 PMC6775525

[B39] Trojnar M. Patro-Małysza J. Kimber-Trojnar Ż. Leszczyńska-Gorzelak B. Mosiewicz J. (2019). Associations between fatty acid-binding protein 4(-)A proinflammatory adipokine and insulin resistance, gestational and type 2 diabetes mellitus. Cells 8 (3), 227. 10.3390/cells8030227 30857223 PMC6468522

[B40] Tscheschner H. Meinhardt E. Schlegel P. Jungmann A. Lehmann L. H. Müller O. J. (2019). CaMKII activation participates in doxorubicin cardiotoxicity and is attenuated by moderate GRP78 overexpression. PLoS One 14 (4), e0215992. 10.1371/journal.pone.0215992 31034488 PMC6488194

[B41] Wang C. Jin H. Wang N. Fan S. Wang Y. Zhang Y. (2016). Gas6/Axl Axis contributes to chemoresistance and metastasis in breast cancer through akt/GSK-3β/β-catenin signaling. Theranostics 6 (8), 1205–1219. 10.7150/thno.15083 27279912 PMC4893646

[B42] Wang K. Zheng J. Yu J. Wu Y. Guo J. Xu Z. (2020). Knockdown of MMP-1 inhibits the progression of colorectal cancer by suppressing the PI3K/Akt/c-myc signaling pathway and EMT. Oncol. Rep. 43 (4), 1103–1112. 10.3892/or.2020.7490 32323782 PMC7057971

[B43] Wang W. Liu Z. Chen X. Lu Y. Wang B. (2021a). Downregulation of FABP5 suppresses the proliferation and induces the apoptosis of gastric cancer cells through the hippo signaling pathway. DNA Cell Biol. 40 (8), 1076–1086. 10.1089/dna.2021.0370 34160301

[B44] Wang Y. Zhu Y. Pu Z. Li Z. Deng Y. (2021b). Soluble resistance-related calcium-binding protein participates in multiple diseases via protein-protein interactions. Biochimie 189, 76–86. 10.1016/j.biochi.2021.06.006 34153376

[B45] Wen L. Liang C. Chen E. Chen W. Liang F. Zhi X. (2016). Regulation of Multi-drug Resistance in hepatocellular carcinoma cells is TRPC6/Calcium Dependent. Sci. Rep. 6, 23269. 10.1038/srep23269 27011063 PMC4806320

[B46] Wen N. Lv Q. Du Z. G. (2020). MicroRNAs involved in drug resistance of breast cancer by regulating autophagy. J. Zhejiang Univ. Sci. B 21 (9), 690–702. 10.1631/jzus.B2000076 32893526 PMC7519632

[B47] Ye Z. Fang B. Pan J. Zhang N. Huang J. Xie C. (2017). miR-138 suppresses the proliferation, metastasis and autophagy of non-small cell lung cancer by targeting Sirt1. Oncol. Rep. 37 (6), 3244–3252. 10.3892/or.2017.5619 28498463 PMC5442395

[B48] Zhang C. Liao Y. Liu P. Du Q. Liang Y. Ooi S. (2020). FABP5 promotes lymph node metastasis in cervical cancer by reprogramming fatty acid metabolism. Theranostics 10 (15), 6561–6580. 10.7150/thno.44868 32550890 PMC7295046

[B49] Zhang J. Guan W. Xu X. Wang F. Li X. Xu G. (2021c). A novel homeostatic loop of sorcin drives paclitaxel-resistance and malignant progression via Smad4/ZEB1/miR-142-5p in human ovarian cancer. Oncogene 40 (30), 4906–4918. 10.1038/s41388-021-01891-6 34163033 PMC8321900

[B50] Zhang M. Wang D. Xu X. Xu W. Zhou G. (2021b). iTRAQ-based proteomic analysis of duck muscle related to lipid oxidation. Poult. Sci. 100 (4), 101029. 10.1016/j.psj.2021.101029 33662660 PMC7937752

[B51] Zhang M. Wang Y. Jiang L. Song X. Zheng A. Gao H. (2021a). LncRNA CBR3-AS1 regulates of breast cancer drug sensitivity as a competing endogenous RNA through the JNK1/MEK4-mediated MAPK signal pathway. J. Exp. Clin. Cancer Res. 40 (1), 41. 10.1186/s13046-021-01844-7 33494806 PMC7830819

[B52] Zheng R. Jia J. Guan L. Yuan H. Liu K. Liu C. (2020). Long noncoding RNA lnc-LOC645166 promotes adriamycin resistance via NF-κB/GATA3 axis in breast cancer. Aging (Albany NY) 12 (10), 8893–8912. 10.18632/aging.103012 32461377 PMC7288957

