# Supplementary material for: Doxorubicin resistance in breast cancer is mediated via the activation of FABP5/PPARγ and CaMKII signaling pathway
Source: Front Pharmacol. 2023 Jul 19;14:1150861. doi: 10.3389/fphar.2023.1150861 (PMC10395833; doi:10.3389/fphar.2023.1150861)
Supplement: Supplementary file 1 [file DataSheet1.DOCX]

Supplementary Material

Dox resistance in breast cancer is mediated via the activation of FABP5/PPARγ and CaMKII signaling pathway

Nan-nan Chen^1^, Bo-ye Han^1^, Xiang-mei Zhang^3^, Zhuang Miao^1^, Ahmed Al-maamari^1^, Jia-min Huang^1^, Yun-jiang Liu^2^, Su-wen Su^1*^

*** Correspondence:** Su-wen Su: suswmk@hebmu.edu.cn

# Supplementary Figures and Tables

## Supplementary Figures


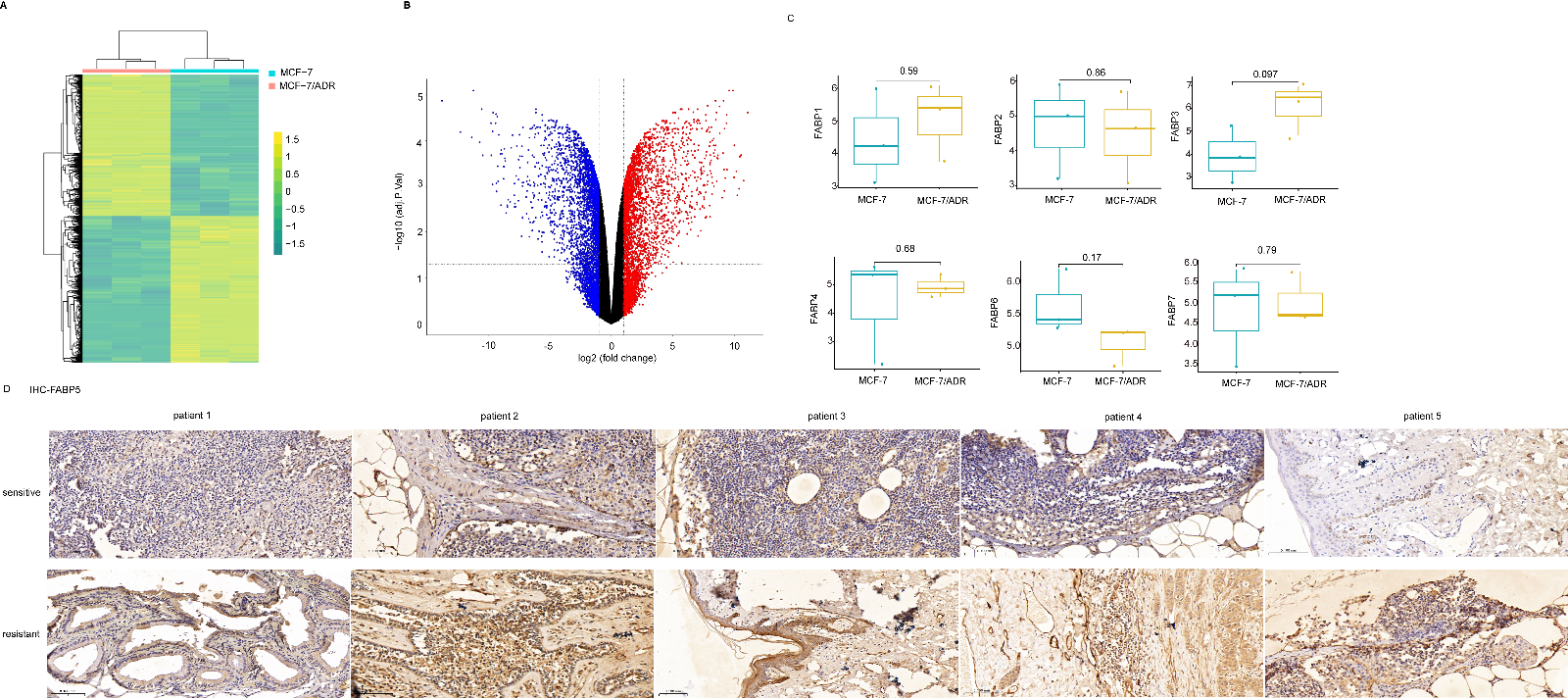


FigS1. FABPs expression in the database and cancer patients. (A)(B) Gene chip analysis of RNA transcripts between MCF-7 cells and MCF-7/ADR cells in the GSE76540 (n=3). (C) Expression box for FABPs according to the GSE76540 dataset. (D) FABP5 expression observed immunohistochemically in tumor patients.


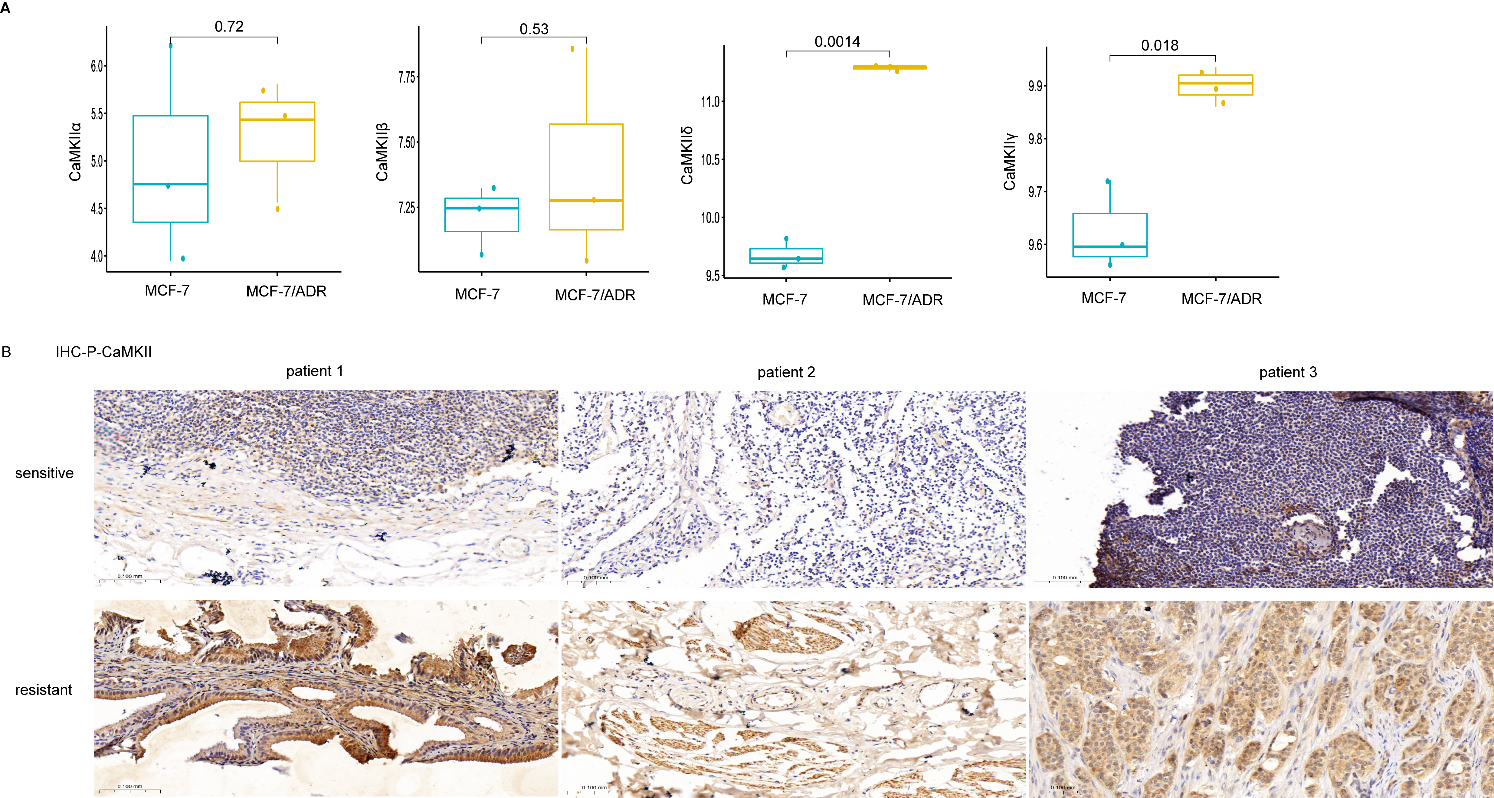


FigS2. CaMKIIs expression in the database and cancer patients. (A) Expression box for CaMKIIs according to the GSE76540 dataset. (B) P-CaMKII expression observed immunohistochemically in tumor patients.


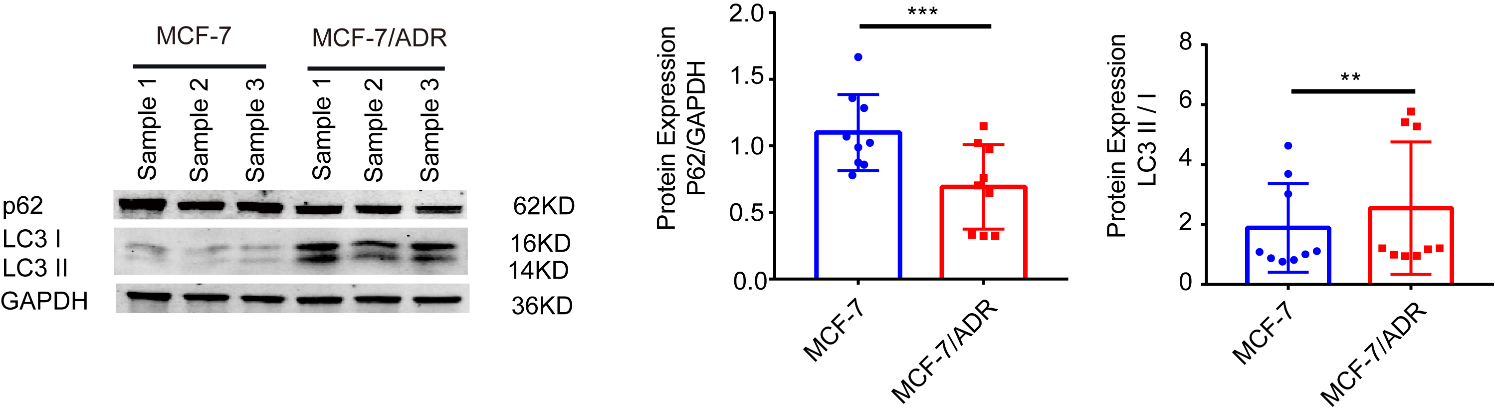


Fig.S3. P62 and LC3B proteins were examined by WB in MCF-7 cells and MCF-7/ADR cells.


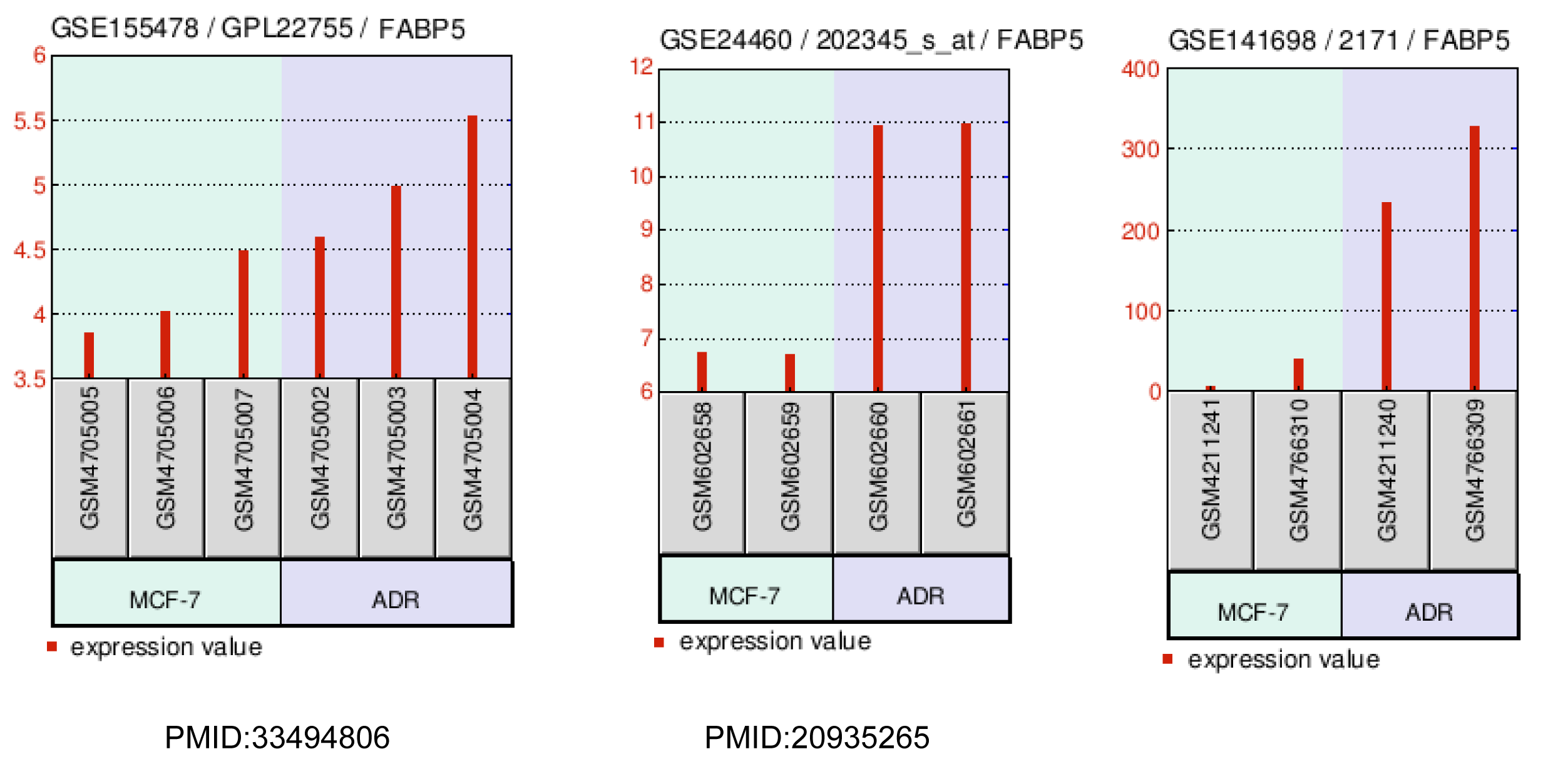


Fig.S4 Expression of FABP5 in GEO datasets (GSE155478, GSE24460, and GSE141698).
